# The Effect of Question Order on Outcomes in the Core Outcome Set for Brief Alcohol Interventions Among Online Help-Seekers: Protocol for a Factorial Randomized Trial

**DOI:** 10.2196/24175

**Published:** 2020-11-26

**Authors:** Marcus Bendtsen, Claire Garnett, Paul Toner, Gillian W Shorter

**Affiliations:** 1 Department of Health, Medicine and Caring Sciences Linköping University Linköping Sweden; 2 Department of Behavioural Science and Health University College London London United Kingdom; 3 Centre for Improving Health-Related Quality of Life School of Psychology Queen’s University Belfast Belfast United Kingdom

**Keywords:** order effects, question order bias, brief alcohol intervention, outcomes, factorial trial, randomized trial, online intervention, alcohol, protocol, effectiveness, efficacy

## Abstract

**Background:**

A core outcome set (COS) for trials and evaluations of the effectiveness and efficacy of alcohol brief interventions (ABIs) has recently been established through international consensus to address the variability of outcomes evaluated.

**Objective:**

This is a protocol for studies to assess if there are order effects among the questions included in the COS.

**Methods:**

The 10 items of the COS are organized into 4 clusters. A factorial design will be used with 24 arms, where each arm represents 1 order of the 4 clusters. Individuals searching online for help will be asked to complete a questionnaire, and consenting participants will be randomized to 1 of the 24 arms (double-blind with equal allocation). Participants will be included if they are 18 years or older. The primary analyses will (1) estimate how the order of the clusters of outcomes affects how participants respond and (2) investigate patterns of abandonment of the questionnaire.

**Results:**

Data collection is expected to commence in November 2020. A Bayesian group sequential design will be used with interim analyses planned for every 50 participants completing the questionnaire. Data collection will end no more than 24 months after commencement, and the results are expected to be published no later than December 2023.

**Conclusions:**

Homogenizing the outcomes evaluated in studies of ABIs is important to support synthesis, and the COS is an important step toward this goal. Determining whether there may be issues with the COS question order may improve confidence in using it and speed up its dissemination in the research community. We encourage others to adopt the protocol as a study within their trial as they adopt the ORBITAL (Outcome Reporting in Brief Intervention Trials: Alcohol) COS to build a worldwide repository and provide materials to support such analysis.

**Trial Registration:**

ISRCTN Registry ISRCTN17954645; http://www.isrctn.com/ISRCTN17954645

**International Registered Report Identifier (IRRID):**

PRR1-10.2196/24175

## Introduction

Alcohol brief interventions (ABIs) have been widely used, researched, and disseminated over the past 60 years [1,2], in both face-to-face [3-5] and digital [6-8] settings and in a variety of populations such as primary care patients [5], emergency health care patients [9,10], college students [11,12], and veterans [13]. Defined by the World Health Organization (WHO) as “practices that aim to identify a real or potential alcohol problem and motivate an individual to do something about it” [3], ABIs encompass a broad range of actions that aim to help individuals change their drinking behavior. At their core, ABIs assess and provide feedback on alcohol use, and can be delivered as a single session or multiple sessions over time, designed to motivate and encourage alcohol change [1,2].

However, the variety of outcome measures used in trial evaluations of ABIs’ effectiveness and efficacy is a limiting factor in evidence synthesis across all modes of intervention delivery (eg, face-to-face and online). Comparisons across trials and synthesis of outcomes as evidence are sometimes impossible despite the use of similar interventions. The ORBITAL (Outcome Reporting in Brief Intervention Trials: Alcohol) project [14] was established to overcome this issue through the determination of an international, consensus-derived core outcome set (COS). The aim was to prioritize the key outcomes to be measured in all online, digital, and otherwise delivered ABIs designed for adult drinkers who are at risk or currently experiencing harm, but who are not seeking treatment. This consensus was derived using the established COMET (Core Outcome Measures in Effectiveness Trials) Methodology [15], including a systematic review that quantified the diversity in outcomes and measurement reporting on 2641 different outcomes, measured in 1560 different ways, in 405 trials of ABIs [16]. This was followed by two e-Delphi (online method to reach consensus) rounds [17], a consensus meeting, and psychometric evaluation to decide the final COS and how outcomes should be measured [18]. The COS established 10 outcomes, which are (1) frequency of drinking, (2) typical number of drinks consumed on a drinking day, (3) frequency of heavy episodic drinking, (4) combined consumption measure, (5) hazardous or harmful drinking, (6) standard drinks consumed in the past week, (7) alcohol-related consequences, (8) alcohol-related injury, (9) use of emergency health care services, and (10) quality of life.

The first 5 outcomes in the COS are measured using the WHO’s Alcohol Use Disorders Identification Test - Consumption (AUDIT-C) tool [19]. Outcome 4 is measured by the total AUDIT-C score, and for outcome 5 a clearly outlined and justified cutoff point of the AUDIT-C score suitable for the country and population should be used. Outcome 6 is measured by asking how many standard drinks were consumed each day of the last week and reported in grams to allow for intercountry comparison.

Alcohol-related problems or consequences (outcome 7) are measured using the Short Inventory of Problems [20,21], with a 3-month time frame. Outcome 8 is measured by asking a single question about injuries inflicted while drinking or being intoxicated, and outcome 9 is similarly measured by a single question about the number of visits to an emergency room or urgent care treatment facility. Finally, quality of life (outcome 10) is measured using PROMIS (Patient-Reported Outcomes Measurement Information System) global health items [22].

This is the first Question Order Bias Core Outcome Set (QOBCOS-1) study, which aims to assess if there is question order bias among the outcomes of the COS. Question order bias occurs when an individual’s response to a question is affected by previously asked questions, and is a well-known phenomenon that has been studied, and perhaps abused, in marketing and political science for some time [23,24]. Recently, it was discovered that question order bias may affect measures of alcohol consumption [25], as individuals who were asked to first report weekly alcohol consumption were then less likely to be screened as risky drinkers, in comparison to individuals who were first screened and then asked about weekly alcohol consumption. However, these findings conflict with previous research that found no evidence of such order effects [26]. Further investigation into this phenomenon is therefore necessary in order to provide better guidance on this potential bias.

This protocol contains the relevant SPIRIT (Standard Protocol Items: Recommendations for Interventional Trials) items [27] and describes a trial that aims to estimate order effects among the questions within the COS for ABIs. In addition, this trial will investigate patterns of abandonment of the questionnaire, as including questions that participants find less relevant may lead to increased attrition [28]. The trial findings will apply in the context of self-completion of the COS using digital questionnaires among online help-seeking individuals. We encourage others to contact the lead author and replicate this protocol in their studies, so that we can collect data for a meta-analysis across different contexts and with different interventions (with due credit).

## Methods

### Trial Design and Interventions

A double-blind randomized factorial design trial will be employed to investigate question order bias among the outcomes of the COS for ABIs. The 10 COS outcomes will be divided into 4 clusters [18]: (1) *average drinking measures:* frequency of drinking, typical number of drinks consumed on a drinking day, frequency of heavy episodic drinking, combined summary consumption measure, hazardous or harmful drinking; (2) *recent drinking measures:* standard drinks consumed in the past week; (3) *quality of life:* health-related quality of life; and (4) *alcohol problems:* alcohol-related problems or consequences, alcohol-related injury, use of emergency health care services.

The order of these clusters will be permuted to create 24 order combinations (Table 1).

**Table 1:** Order combinations of the 4 item clusters creating 24 trial arms.

Arm	Cluster 1	Cluster 2	Cluster 3	Cluster 4
1	1	2	3	4
2	1	2	4	3
3	1	3	2	4
4	1	3	4	2
5	1	4	2	3
6	1	4	3	2
7	2	1	3	4
8	2	1	4	3
9	2	3	1	4
10	2	3	4	1
11	2	4	1	3
12	2	4	3	1
13	3	1	2	4
14	3	1	4	2
15	3	2	1	4
16	3	2	4	1
17	3	4	1	2
18	3	4	2	1
19	4	1	2	3
20	4	1	3	2
21	4	2	1	3
22	4	2	3	1
23	4	3	1	2
24	4	3	2	1

### Setting and Participants

**Figure 1:**
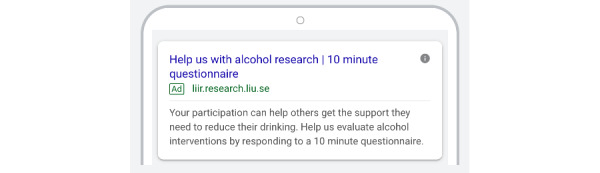
Example of online advertisement used to recruit trial participants.

This trial received ethical approval on July 1, 2020, from the Swedish Ethical Review Authority (Dnr 2020-01799). English-speaking individuals searching online for information on how to drink less or quit drinking will be recruited using Google Ads with language targeting. Language targeting is an automated process in which Google’s algorithms will display the advert to individuals using their products (eg, search and Gmail) in the specified language. Examples of search queries targeted are “How do I drink less,” “I drink too much,” and “Support for drinkers.” The recruitment information will be framed as an invitation to take part in a study that aims to improve alcohol intervention research, and it will be made clear that participants should not expect to receive support. An example of an advert is shown in Figure 1, and study information presented to individuals who click on the advert can be found in Multimedia Appendix 1.

Individuals will be asked to read the study information presented when the advert is clicked on and confirm that they are at least 18 years old and consent to take part in the trial (see Multimedia Appendix 1). Participants consenting to take part in the trial will be randomized to one of the arms of the trial (Table 1). Thus, there will be no explicit exclusion criteria; however, analyses will exclude participants reporting having not consumed any alcohol during the past 3 months (ie, answering *Never* to the first AUDIT-C question and having consumed 0 drinks in the past week). Questions will be presented to participants in the order that corresponds to their group allocation. Participants will be allowed to go back and change their responses to previous questions (to make the experiment similar to regular surveys and trials; see Discussion). Once all questions have been answered, participants will be thanked and recommended to read more about alcohol and health on a selection of websites. No further contact will be made with the participants.

### Outcomes

The primary outcomes are the 10 outcomes of the COS measured using the recommended questionnaires [18], and the proportion of participants abandoning the questionnaire.

The secondary outcomes are the proportion of participants visiting the provided links at the end of the questionnaire, and time spent on the questionnaire among completers and abandoners.

The first primary outcome (ie, the responses to the COS) will facilitate the primary analysis of question order effects. As the COS is new, we also wish to measure the abandonment rate in order to guide both future trials utilizing the COS and further development of the COS for online and digital settings.

Measuring the proportion of participants visiting the provided links at the end of the questionnaire is an opportunistic decision to gather some data on the degree to which responding to the COS satisfies participants’ intentions to seek help online. Assessment has been found to affect alcohol outcomes [11,29,30]; thus, differentiation between those who visit the links at the end of the questionnaire with respect to responses to the COS will generate hypotheses for future trials aimed at understanding who is affected by the assessment. The final outcome, time spent on the questionnaire, is captured primarily to guide future research on the anticipated participant burden of completing the COS.

### Randomization and Blinding

Block randomization (random block sizes of 24 and 48) will be used to achieve equal allocation among arms. The randomization sequence and allocation will be fully automated and computerized. Since no identifiers are collected for individuals, we will use web browser cookies and HTML5 storage to store allocation information on the participants’ web browsers (see Discussion). Participants who have not completed the questionnaire and return to the trial website will be presented with the cluster order according to their assignment. Participants who have completed the questionnaire and return to the trial website will be thanked for their participation, but not offered an opportunity to answer the questions again.

Participants will be aware that they are taking part in a research study; however, the true nature of the study will not be revealed to them, since this would interfere with the effect being studied. Therefore, participants will not be aware of which arm they are in, and hence will be blinded to allocation. Researchers will also be blind to participant allocation.

### Analysis

#### Preliminary

All analyses will be conducted according to intention-to-treat principles, with all participants analyzed in the groups to which they were randomized. Analyses will exclude participants who report not having consumed any alcohol the past 3 months (ie, answering *Never* to the first AUDIT-C question and having consumed 0 drinks in the past week). Analyses will initially be done using complete cases, and sensitivity analyses with imputed values will be used to assess robustness of results under different assumptions of the missing data. Estimates for model parameters will be interpreted by inspecting marginal posterior distributions using Bayesian inference (see Sample Size for prior specification) [31-33] and complemented by null hypothesis testing (at the .05 significance level).

#### Primary Analysis

The primary analysis of question order bias will be conducted through regression models in which each outcome in each cluster will be regressed against a dummy variable representing whether each of the other clusters was asked before or after the outcome. For instance, standard drinks consumed in the past week (which is part of Cluster 2), will be regressed against 3 dummy variables, representing Cluster 1, Cluster 3, and Cluster 4, respectively. The dummy variables will take value 0 if the cluster was asked after Cluster 2 and value 1 if the cluster was asked before Cluster 2. For each outcome, 1 regression model will be created, yielding a total of 10 models, using negative binomial regression for counts (outcome 6 and outcome 9), logistic regression for hazardous or harmful drinking (outcome 5, using AUDIT-C scores of 5+ as the cutoff), and normal regression for scores (all other outcomes, possibly log-transformed if found to be skewed).

We will investigate 2- and 3-way interactions among the cluster dummy variables in order to explore if the order of a combination of clusters affects outcomes (eg, if the order of Cluster 1 and Cluster 2 in combination creates a question order bias on the outcomes in Cluster 3).

The proportion of participants abandoning the questionnaire will be analyzed in two ways: (1) using a logistic regression model with allocated arm as a covariate, to identify orders that are more (or less) likely to result in abandonment, and (2) using a logistic regression model with the cluster that was abandoned and the number of questions responded to as covariates, to identify clusters that are more (or less) likely abandoned (adjusted for number of questions responded to).

#### Secondary Analysis

The proportion of participants visiting the provided links at the end of the questionnaire will be analyzed using a logistic regression model with the COS outcomes as covariates under both standard normal priors and shrinkage priors [34].

Time spent (in seconds) on the questionnaire will be reported among completers and abandoners and analyzed in two ways: (1) using normal regression with arm as a covariate (completers and abandoners, possibly log-transformed) and (2) using normal regression with the COS outcomes as covariates (completers only, possibly log-transformed). Both analyses will be conducted under standard normal priors, and the second analysis will also be conducted using shrinkage priors [34].

#### Exploratory Analysis

Patterns among individuals with respect to going back and changing responses to previous questions will be investigated in exploratory analyses using a combination of regression and clustering models. We will also run sensitivity analyses to see if the primary findings change when using the first response option that participants chose.

### Sample Size

The trial will use a Bayesian group sequential design [35-37] to monitor recruitment, with interim analyses planned for every 50 participants completing the questionnaire. Responses to each of the 10 COS outcomes will be modelled following the primary analyses, and each dummy variable representing cluster order will be assessed for evidence of effect or futility. Let ß_k,i_ represent the coefficients for each dummy variable (i=1,2,3) in each model (k=1...10) and D represent the data available at the interim analyses. Then, the target criteria will be (1) *effect:* p(ß_k,i_ > 0 | D) > 97.5% or p(ß_k,i_ < 0 | D) > 97.5% (ie, if the question order effect is greater or less than 0 with a probability greater than 97.5%); (2) *futility (normal regression):* p(−0.1 < ß_k,i_ < 0.1 | D) > 95% (ie, if the question order effect is close to 0 with a probability greater than 95%); and (3) *futility (negative binomial and logistic regression):* p(log(1/1.2) < ß_k,i_ < log(1.2)) > 95% (ie, if the question order effect is close to 0 with a probability greater than 95%).

For the effect criterion, we will use a skeptical normal prior for dummy covariates (mean 0, SD 1.0), and a wider prior will be used for the futility criterion (mean 0, SD 2.0).

The criteria should be viewed as targets; thus, at each interim analysis, we will evaluate each criterion for each covariate and decide if we believe that recruitment should end. We will only make decisions to stop recruitment entirely, not drop or modify any of the arms. Simulations indicate that we will require a sample size in the range of 1500 to 2500 participants. Recruitment will not exceed 24 months.

## Results

Recruitment will commence in November 2020. Findings from this study are expected to be disseminated in peer-reviewed journals and presented at relevant international conferences during 2021-2023, after which all data will be made available on the Open Science Framework. Protocols and standard operating procedures will be developed to promote replication, including in modes other than online, and all models will be hosted on the Open Science Framework [38].

## Discussion

### Overview

This study will be the first to assess question order bias in the COS for ABIs, and will help guide future trials in how to ask the COS. It is clear that the research field as a whole would benefit from reducing heterogeneity in the outcomes used in trials; thus, the findings from this study may help increase confidence in using the COS.

### Limitations and Generalizability

Respondents will be able to go back and change their responses to previous questions as they progress through the items; if the aim of the study was to capture causal connections among constructs represented by the clusters, a method used in other studies [39], then this process of changing responses would have been inappropriate. However, the aim of the study is to capture question order bias among the items as they would be used in a regular survey or trial; thus, not allowing participants to change previous responses would reduce generalizability. However, if this trial finds evidence of question order bias, then future trials should test not allowing previous responses to be changed with the aim of testing causal connections.

There is no reason to collect and verify any unique identifiers or means of contact for each participant (eg, phone number of email), since this trial does not require any follow-up. This, however, also means that there is no way of connecting group allocation to such a unique identifier. Instead, we will use HTML5 storage and cookies in participants’ web browsers to store group allocation information, such that when participants return to the study website, they will not be rerandomized. However, participants could be rerandomized if they join using a different computer or web browser. This is a limitation of this trial that we find necessary in order to retain interested individuals in the trial, as confirming email addresses and phone numbers would increase participant burden and reduce the participation rate. We will, however, keep track of the number of times each participant visits our website using the same device. A high rate of return from the same device would increase the likelihood that participants also visit from other devices, and vice versa. Therefore, we can use this measure to help judge the risk of bias from double randomization. In addition, the links to websites with alcohol information at the end of the survey aim to satisfy the need of participants to search for this material again, reducing the risk that they revisit the study website.

As is often the case in online studies, participants sign up to the trial on their own. We have no screening questions to exclude participants who are not seeking help with their alcohol consumption, as this may interfere with the study of the order effects. Therefore, some may participate because they are curious about the study or seeking help for others. A single question at the end of the survey (not part of the factorial allocation) will explore participants’ intentions (“Was the aim of your participation in this study to get help to reduce your alcohol use?”). We will also analyze the search strings used by those clicking on the advert to capture the intentions of the study sample. We will not, however, make any adjustments to our primary analyses, but rather consider this uncertainty a limitation of generalizability of the findings of the trial.

Other limitations of this trial include clustering of certain outcomes in the COS. Alternative clustering may reveal different findings, and it would also be possible to randomize the order of each question without clusters; however, the number of arms would exceed what is feasible for factorial trials. The findings may also not apply to modes other than online data collection (eg, order effects may not hold in paper-based or face-to-face administration).

## Appendix

Multimedia Appendix 1 Informed consent materials.

## Abbreviations

ABI: alcohol brief intervention
AUDIT-C: Alcohol Use Disorders Identification Test – Consumption
COMET: Core Outcome Measures in Effectiveness Trials
COS: core outcome set
ORBITAL: Outcome Reporting in Brief Intervention Trials: Alcohol
PROMIS: Patient-Reported Outcomes Measurement Information System
QOBCOS-1: Question Order Bias Core Outcome Set
SPIRIT: Standard Protocol Items: Recommendations for Interventional Trials
WHO: World Health Organization
