# Correction: The Effect of Question Order on Outcomes in the Core Outcome Set for Brief Alcohol Interventions Among Online Help-Seekers: Protocol for a Factorial Randomized Trial

**DOI:** 10.2196/26578

**Published:** 2021-02-01

**Authors:** Marcus Bendtsen, Claire Garnett, Paul Toner, Gillian W Shorter

**Affiliations:** 1 Department of Health, Medicine and Caring Sciences Linköping University Linköping Sweden; 2 Department of Behavioural Science and Health University College London London United Kingdom; 3 Centre for Improving Health-Related Quality of Life, School of Psychology Queen’s University Belfast Belfast United Kingdom

In “The Effect of Question Order on Outcomes in the Core Outcome Set for Brief Alcohol Interventions Among Online Help-Seekers: Protocol for a Factorial Randomized Trial” (JMIR Res Protoc 2020;9(11):e24175) the authors noted one error.

The protocol included an appendix which contains questionnaires which have been proposed to measure a core outcome set for brief alcohol interventions. However, rather than including their own appendix, the authors acknowledge that they should have referenced the Open Science Framework project which contains materials for the core outcome set.

The file labelled as Multimedia Appendix 1 in the originally published article has been removed from the corrected version. In-text references to this appendix have been replaced by a citation to Reference 18, which contains a link to the Open Science Framework project. Accordingly, the file labelled as Multimedia Appendix 2 in the originally published article has renamed Multimedia Appendix 1 in the corrected version. In-text references to Multimedia Appendix 2 have been changed to Multimedia Appendix 1. These changes affect the text in the following places:

In the Methods section, under “Trial Design and Interventions”, the sentence:

The 10 COS outcomes will be divided into 4 clusters (for details, please see Multimedia Appendix 1)

has been replaced by:

The 10 COS outcomes will be divided into 4 clusters [18]”

Under “Setting and Participants”, the sentence:

An example of an advert is shown in Figure 1, and study information presented to individuals who click on the advert can be found in Multimedia Appendix 2.

has been changed to:

An example of an advert is shown in Figure 1, and study information presented to individuals who click on the advert can be found in Multimedia Appendix 1.

Likewise, the sentence:

Individuals will be asked to read the study information presented when the advert is clicked on and confirm that they are at least 18 years old and consent to take part in the trial (see Multimedia Appendix 2).

has been changed to:

Individuals will be asked to read the study information presented when the advert is clicked on and confirm that they are at least 18 years old and consent to take part in the trial (see Multimedia Appendix 1).

Under “Outcomes”, the sentence:

The primary outcomes are the 10 outcomes of the COS measured using the recommended questionnaires (Multimedia Appendix 1)

has been changed to:

The primary outcomes are the 10 outcomes of the COS measured using the recommended questionnaires [18]

The correction will appear in the online version of the paper on the JMIR Publications website on February 1, 2021, together with the publication of this correction notice. Because this was made after submission to PubMed, PubMed Central, and other full-text repositories, the corrected article has also been resubmitted to those repositories.

