# Supplementary material for: The Effect of Question Order on Outcomes in the Core Outcome Set for Brief Alcohol Interventions Among Online Help-Seekers: Protocol for a Factorial Randomized Trial
Source: JMIR Res Protoc. 2020 Nov 26;9(11):e24175. doi: 10.2196/24175 (PMC7728524; doi:10.2196/24175)
Supplement: Multimedia Appendix 1 [file resprot_v9i11e24175_app1.docx]

# Multimedia Appendix 2 – Informed Consent materials

**Do you want to participate in the “Alcohol and its consequences” study?**

Thank you for showing interest in this study on alcohol consumption and its consequences. By pressing on the button “I am at least 18 years old and consent to take part in this study” you are consenting to the information below and you will be taken directly to the questionnaire, which will take approximately 10 minutes to complete. Please note that this is only a survey and there will be no support offered to you, instead you will be recommended websites after the survey which you can visit for support.

**Why are we doing this study and why do you want me to participate?** In alcohol research it is common to ask participants to respond to questionnaires regarding their past alcohol consumption. In order for research findings to be accurate, it is important that questionnaires are good at measuring alcohol consumption. This study aims to gain knowledge on creating questionnaires for alcohol research. Since you have searched online for support with alcohol consumption you are part of the group of people that we want to study.

This research project is conducted at Linköping university in Sweden, please find contact details for the primary investigator below.

**What is expected of me?** Your participation is very important for future alcohol research projects, and we are asking you to complete a questionnaire which takes approximately 10 minutes. There are several versions of the questionnaire, and you will be randomly given one of the different versions to fill out.

**Will I be taking any risks by participating in this study?** This study only asks you to respond to a questionnaire, but will not give you any support for behaviour change. If you want to reduce your consumption you should not expect to be helped by participating in this study, but we will give you resources at the end of the questionnaire which you may find helpful. There is a risk that responding to questions without getting any feedback may have a de-motivating effect for change, and you should be aware of this before deciding to take part.

**Will I be leaving any personal information?** We will collect data from you about your alcohol consumption, consequences that you have experienced, and your general physical and mental health. However, we will not collect any personal identifier from you, so all data that we collect will be completely anonymous. The data collection is for scientific research, and is therefore motivated by public interest (GDPR EU 2016/679, Prop. 2017/18:298).

**Where will data be stored and who will have access?** During the study period we will store all data on a database at Linköping university in Sweden. After the study period the data will be archived securely at Linköping university (Sweden) and removed from the database. Access to the data will at first be restricted to the research team, but will later be made publicly available for other researchers to analyse. Please note that all data will be anonymous, and cannot be traced back to you.

**Who is responsible for the study?** Linköping university is responsible for the data collected in this study. Note that since you are not leaving any personal identifier we cannot delete your data after you have responded to the survey, as we cannot identify which data belongs to you. If you have any questions about your data you can contact Dr. Marcus Bendtsen, (marcus.bendtsen@liu.se), or the data protection officer (dataskyddsombudet@liu.se). If you are not satisfied with the way your personal data has been handled, you can file a complaint at the Swedish Data Protection Authority.

**How can I get more information about the results from this study?** All results will be published in peer-reviewed journals and conferences no later than 2022.

**Participation is voluntary.** You can at any point decide to not be part of the study and you will not have to explain why.

**Contact details**Dr. Marcus Bendtsen, marcus.bendtsen@liu.se
Senior Lecturer in Experimental Social Medicine and Public Health
Department of Health, Medicine and Caring Sciences, Linköping University
581 83 Linköping, Sweden
